# Red hybrid tilapia (*Oreochromis* sp.) hindgut RNA-seq data after oral vaccination with a feed-based bivalent vaccine

**DOI:** 10.1016/j.dib.2024.110977

**Published:** 2024-09-26

**Authors:** Nur Shidaa Mohd Ali, Mohamad Syazwan Ngalimat, Mohd Zamri Saad, Mohammad Noor Amal Azmai, Annas Salleh, Zarirah Zulperi, Ina Salwany Md Yasin

**Affiliations:** a Laboratory of Aquatic Animal Health and Therapeutics, Institute of Bioscience, Universiti Putra Malaysia UPM, 43400 Serdang, Selangor, Malaysia; b Department of Microbiology, Faculty of Biotechnology and Biomolecular Sciences, Universiti Putra Malaysia UPM, 43400 Serdang, Selangor, Malaysia; c Department of Veterinary Laboratory Diagnosis, Faculty of Veterinary Medicine, Universiti Putra Malaysia UPM, 43400 Serdang, Selangor, Malaysia; d Department of Biology, Faculty of Science, Universiti Putra Malaysia UPM, 43400 Serdang, Selangor, Malaysia; e Department of Aquaculture, Faculty of Agriculture, Universiti Putra Malaysia UPM, 43400 Serdang, Selangor, Malaysia

**Keywords:** Oreochromis sp., Aeromoniasis, Streptococcosis, Feed-based vaccine, Immuno-transcriptome

## Abstract

Previous studies have proven that red hybrid tilapia (*Oreochromis* sp.) vaccinated with a feed-based bivalent vaccine incorporating the formalin-killed whole organisms *Streptococcus agalactiae* and *Aeromonas hydrophila* mixed with 10 % palm oil showed good protection against streptococcosis and aeromoniasis diseases. However, the molecular mechanisms related to the induction of fish's immunological responses after vaccination are poorly investigated. Therefore, a transcriptomic study using the hindgut of red hybrid tilapia after vaccination was conducted, as the gut plays a role in antigen uptake and nutrient absorption. The transcriptome dataset has the potential to provide an understanding of the early induction of immunological responses in red hybrid tilapia after vaccination. Here, the vaccinated and control red hybrid tilapia's hindgut ribonucleic acid sequencing (RNA-seq) dataset was presented, which are available in the National Center for Biotechnology Information (NCBI) database with accession number PRJNA1014699.

Specifications Table

**Table:** 

Subject	Agricultural Sciences
Specific subject area	Aquaculture and Aquatic Science
Type of data	RNA-seq raw reads, Table, Figure
Data collection	The hindgut of vaccinated and control fish was collected and the total ribonucleic acid (RNA) was extracted according to the previous study [1]. The sample was sequenced using the Illumina HiSeq™ 4000 platform and mapped to the *Oreochromis niloticus* Orenil1.1 genome (GCF000188235v2) for the transcriptomic analysis.
Data source location	Laboratory of Aquatic Animal Health and Therapeutics, Institute of Bioscience, Universiti Putra Malaysia, 43,400 UPM, Serdang, Selangor, Malaysia.
Data accessibility	Repository name: National Center for Biotechnology Information (NCBI) BioProject and Mendeley Data Data identification number: NCBI BioProject (PRJNA1014699) and Mendeley Data (DOI: 10.17632/hxn9539pjp.1) Direct URL to data: https://www.ncbi.nlm.nih.gov/bioproject/PRJNA1014699 and https://data.mendeley.com/datasets/hxn9539pjp/1
Related research article	N.S.M. Ali, M.S. Ngalimat, M.Z. Saad, M.N.A. Azmai, A. Salleh, Z. Zulperi, I.S. Md Yasin, Expression of immuno-transcriptome response in red hybrid tilapia (*Oreochromis* sp.) hindgut following vaccination with feed-based bivalent vaccine, J Fish Dis. 47 (2024) e13943. https://doi.org/10.1111/jfd.13943 [1] N.S. Mohd Ali, M.Z. Saad, M.N.A. Azmai, A. Salleh, Z.M. Zulperi, T. Manchanayake, M.A.D. Zahaludin, L. Basri, A. Mohamad, I.S. Md Yasin, Immunogenicity and efficacy of a feed-based bivalent vaccine against streptococcosis and motile aeromonad septicemia in red hybrid tilapia (*Oreochromis* sp.), Animals. 13 (2023) 1346. https://doi.org/10.3390/ani13081346 [2]

## 1 Value of the Data

• The data represent the first hindgut transcriptomic responses after oral vaccination with a feed-based bivalent vaccine incorporating the formalin-killed whole organisms *S. agalactiae* and *A. hydrophila* mixed with 10% palm oil in red hybrid tilapia.
• The data could provide an understanding of the early induction of immunological responses in red hybrid tilapia after oral vaccination with a feed-based bivalent vaccine.
• The RNA-seq data can be used as a benchmark to identify differentially expressed genes in the response to oral vaccination in red hybrid tilapia's hindgut.

## 2 Background

In Malaysia, the most commonly reported bacterial infection in red hybrid tilapia (*Oreochromis* sp.) culture is caused by pathogenic bacteria from the genera *Streptococcus* spp. and *Aeromonas* spp. [3]. A feed-based vaccine with the incorporation of formalin-killed whole organism *S. agalactiae* into the feed with 10 % of palm oil as an adjuvant has improved fish immunity against bacterial infection [4]. In 2023, a feed-based bivalent vaccine incorporating the formalin-killed whole organisms *S. agalactiae* and *A. hydrophila* mixed with 10 % palm oil has been developed [2]. Fish vaccinated with a feed-based bivalent vaccine recorded relative percentage survival (RPS) at 90 % when challenged with pathogenic *A. hydrophila*, followed by *S. agalactiae* (RPS at 80 %), *S. iniae* (RPS at 63 %) and *A. veronii* (RPS at 60 %) [2]. Additionally, vaccinated fish showed significant (*p* ≤ 0.05) improvement in innate and adaptive immunological responses as indicated based on the expression of immune-related genes [1] as well as lysozyme and immunoglobulin M (IgM) productions [2]. To further investigate the molecular mechanisms related to the induction of fish's immunological responses after vaccination, the fish's hindgut was subjected to RNA-sequencing (RNA-seq), as the gut plays a role in antigen uptake and nutrient absorption [5,6].

## 3 Data Description

The transcriptomic response after oral vaccination with a feed-based bivalent vaccine incorporating the formalin-killed whole organisms *S. agalactiae* and *A. hydrophila* mixed with 10 % palm oil in the red hybrid tilapia's hindgut was investigated. The transcriptomic response was compared with that of unvaccinated (control) fish [1]. The FASTQ RNA-seq raw data file of vaccinated and unvaccinated fish has been deposited in the NCBI database under the BioProject accession number PRJNA1014699. The dataset provides a benchmark for understanding the early induction of immunological responses in red hybrid tilapia's hindgut after oral vaccination. The descriptive information for RNA-seq raw data generated from vaccinated and unvaccinated fish's hindguts is given in Table 1. Briefly, the vaccinated and control fish hindguts’ datasets produced a total of 224,487,888 base pair (bp) and 190,615,084 bp of clean reads respectively, accounting for an average ratio of 98.95 % (vaccinated) and 99.00% (control) of raw reads after removing adaptor sequences and low-quality reads. The average Q20, Q30 and guanine-cytosine (GC) content ratios for clean reads were 97.64 %, 93.21 % and 47.96 %, respectively. Raw data from this work have also been uploaded to the Mendeley Data and can be accessed at https://data.mendeley.com/datasets/hxn9539pjp/1.

**Table 1:** The descriptive information for RNA-seq raw data generated from of vaccinated and unvaccinated fish's hindgut.

Descriptive	Sample			
	V1	V2	C1	C2
Library ID	DRRA220009877-1a	DRRA220009878-1a	DRRA220009875-1a	DRRA220009876-1a
Total raw reads (Mb)	114.78	112.09	94.81	97.73
Total clean reads (Mb)	113.62	110.87	93.98	96.63
Clean read Q20 (%)	97.35	97.81	97.71	97.68
Clean read Q30 (%)	92.54	93.61	93.37	93.30
Guanine-Cytosine content (%)	47.81	47.91	48.92	47.20
Clean read ratio (%)	87.14	84.61	85.71	86.64
SRA accession number	SRX21711784	SRX21711851	SRX21709845	SRX21711756
BioSample accession number	SAMN43270085	SAMN43270096	SAMN37338787	SAMN43270072

Sample V1: The replicate one of total RNA extracted from the vaccinated fish's hindgut.

Sample V2: The replicate two of total RNA extracted from the vaccinated fish's hindgut.

Sample C1: The replicate one of total RNA extracted from the unvaccinated (control) fish's hindgut.

Sample C2: The replicate two of total RNA extracted from the unvaccinated (control) fish's hindgut.

Total raw reads (Mb): The reads amount before filtering.

Total clean reads (Mb): The reads amount after filtering.

Clean read Q20 (%): The rate of bases which quality is greater than 20 value in clean reads.

Clean read Q30 (%): The rate of bases which quality is greater than 30 value in clean reads.

Clean read ratio (%): The ratio of the amount of clean reads.

## 4 Experimental Design, Materials and Methods

In this study, the feed-based bivalent vaccine was generated by mixing the formalin-killed whole organisms *S. agalactiae* strain SA2k and *A. hydrophila* strain Ah1Sa5 with 10 % (v/w) food-grade palm oil (Yee Lee Edible Oils Sdn. Bhd., Malaysia) and commercial tilapia feed (Star Feedmills (M) Sdn. Bhd., Malaysia) powder according to the Malaysia Intellectual Property Corporation patent number PI20222001807. Meanwhile, the feed-based bivalent vaccine prepared without the addition of bacteria was used as a control. Prior to vaccination, fish were starved for 24 h. The fish vaccination study was conducted according to previous studies [1,2]. Briefly, red hybrid tilapia (*n* = 60) were separated into two groups (vaccinated and control), and each group contained 30 fish/group. The vaccines were delivered at 5 % fish body weight for three consecutive days on week 0, followed by booster vaccination on weeks 2 and 6. Fish were fed with commercial tilapia feed (Star Feedmills (M) Sdn. Bhd., Malaysia) twice daily for the nonvaccination days.

**Fig. 1:**
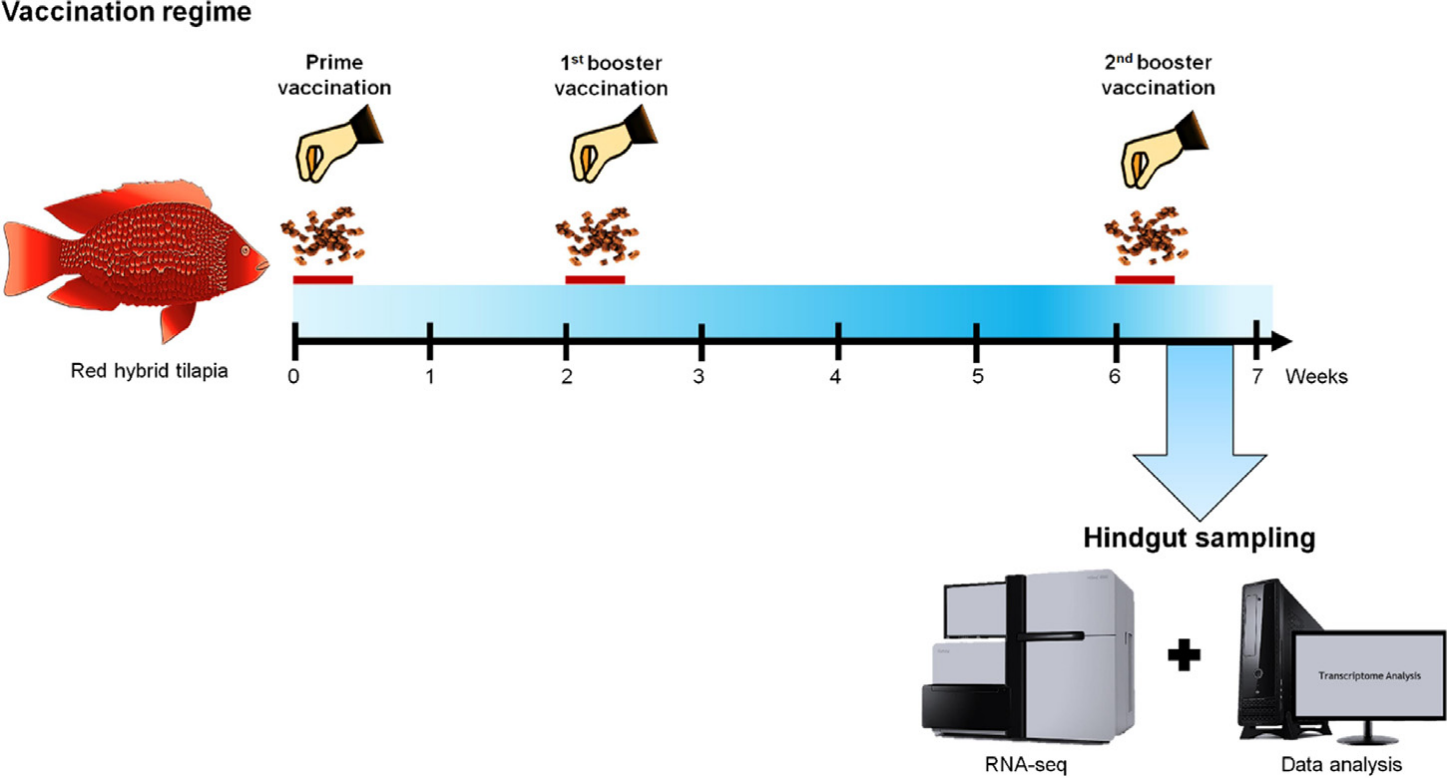
Timeline of the vaccination regime and sample collection for transcriptomic analysis. The feed-based vaccine was delivered at 5 % of the fish's body weight for three consecutive days (red bar) in weeks 0 (prime vaccination), 2 (1st booster vaccination), and 6 (2nd booster vaccination). For hindgut sampling, hindgut samples were collected after the 2nd booster vaccination at week 6 at 48 h’ post-vaccination.(For interpretation of the references to color in this figure legend, the reader is referred to the web version of this article.)

The hindgut samples from vaccinated and control fish were collected (Fig. 1) according to the previous study [1]. The total RNA was extracted from the hindgut samples (pooled from 3 fish/replicate for each vaccinated and control fish) using TRIzol reagent according to the manufacturer's instructions (Invitrogen, USA). A total of 1 µg of total RNA/sample was used for complementary DNA (cDNA) library construction following the protocol supplied with the NEBNext Ultra™ RNA Library Prep Kit for Illumina (NEB, USA). The amplified cDNA fragments were sequenced by the Illumina HiSeq™ 4000 platform (Illumina, USA), where 240 bp paired-end reads were generated. Raw reads in FASTQ format were first processed through in-house Perl scripts. To obtain clean reads, reads that contain adapter sequences, reads with ambiguous “N” and low-quality reads (>50 % of reads with a quality score Q-value ≥ 20) were removed from the raw reads. The ratio of clean read Q20, Q30 and GC content were calculated, and all the downstream analyses relied on the clean reads. After removing ribosomal RNA using the short-read alignment tool Bowtie2 (version 2.2.8) [7,8], paired-end clean reads were mapped to the *Oreochromis niloticus* Orenil1.1 genome (GCF000188235v2) using TopHat2 (version 2.0.3.12) [9]. The mapped reads of each sample were assembled using StringTie (version 1.3.0) [10]. Raw read files were submitted to the Sequence Read Archive (SRA) with accession numbers SRX21711784 (vaccinated replicate 1), SRX21711851 (vaccinated replicate 2), SRX21709845 (control replicate 1), SRX21711756 (control replicate 2) and linked to BioProject PRJNA1014699 on the NCBI databases. The raw read file also linked to the BioSample with accession numbers SAMN43270085 (vaccinated replicate 1), SAMN43270096 (vaccinated replicate 2), SAMN37338787 (control replicate 1) and SAMN43270072 (control replicate 2).

## Limitations

Not applicable.

## Ethics Statement

All procedures in this study involving animals were performed following the Department of Biosafety, Ministry of Natural Resources and Environment, Malaysia. The ethic was approved by the Institutional Animal Care and Use Committee, Universiti Putra Malaysia (UPM) with the approval number of UPM/IACUC/AUP-R076/2019.

## CRediT Author Statement

**Ina Salwany Md Yasin**: Conceptualization; Data curation; Formal analysis; Investigation; Methodology; Funding acquisition; Project administration; Supervision; Resources; Validation; Writing - Review & Editing. **Nur Shidaa Mohd Ali**: Conceptualization; Data curation; Formal analysis; Investigation; Methodology; Validation; Visualization; Writing-Original Draft; Writing - Review & Editing. **Mohamad Syazwan Ngalimat**: Conceptualization; Data curation; Formal analysis; Investigation; Methodology; Visualization; Writing-Original Draft; Writing - Review & Editing. **Mohd Zamri Saad**: Funding acquisition; Project administration; Supervision. **Mohammad Noor Amal Azmai**: Funding acquisition; Project administration; Supervision; Writing - Review & Editing. **Annas Salleh**: Funding acquisition; Project administration; Supervision. **Zarirah Zulperi**: Project administration; Supervision.

## Data Availability

Red hybrid tilapia (Oreochromis sp.) hindgut RNA-seq data after oral vaccination with a feed-based bivalent vaccine (Original data) (Mendeley Data). Induction of immunological expression in red hybrid tilapia (Oreochromis sp.) hindgut following vaccination with feed-based bivalent vaccine (Original data) (National Center for Biotechnology Information (NCBI)).
